# O‐Arm Navigated Cervical Pedicle Screw Fixation in the Treatment of Lower Cervical Fracture‐Dislocation

**DOI:** 10.1111/os.13227

**Published:** 2022-05-07

**Authors:** Kai Zhang, Hao Chen, Kangwu Chen, Peng Yang, Huilin Yang, Haiqing Mao

**Affiliations:** ^1^ Department of Orthopedic Surgery The First Affiliated Hospital of Soochow University Suzhou China; ^2^ Department of Orthopedic Surgery Affiliated Hospital of Yangzhou University Yangzhou China; ^3^ Institute of Translational Medicine Medical College, Yangzhou University Yangzhou China

**Keywords:** Cervical pedicle screw, Navigation, O‐arm

## Abstract

**Objective:**

To evaluate the safety and efficacy of cervical pedicle screw (CPS) placement with O‐arm navigation in the treatment of lower cervical fracture‐dislocation.

**Methods:**

A retrospective clinical study was performed involving 42 consecutive patients with lower cervical spine fracture‐dislocation who underwent CPS fixation surgery with O‐arm navigation (CPS group) or received conventional lateral mass screw (LMS) fixation surgery (LMS group) between August 2015 and August 2019. Accuracy of CPS position was evaluated by postoperative CT. The clinical parameters including preoperative and final follow‐up Japanese Orthopaedic Association (JOA) score and American Spinal Injury Association (ASIA) Impairment Scale, preoperative Sub‐axial Injury Classification (SLIC) score, number of fixation segments, operation time, intraoperative blood loss, injury mechanism, injury location, surgical complications were also assessed between the two groups.

**Results:**

In LMS group, the preoperative SLIC score was 7.5 ± 0.9, ASIA score improvement was 0.8 ± 0.5, JOA score improvement was 3.0 ± 1.8, mean operation time was 204 ± 89 min, intraoperative blood loss was 311 ± 127 ml. In CPS group, the preoperative SLIC score was 7.3 ± 1.2, ASIA score improvement was 0.9 ± 0.5, JOA score improvement was 3.2 ± 2.4, mean operation time is 241 ± 85 min, intraoperative blood loss is about 327 ± 120 ml. There was no significant difference in terms of above clinical parameters between the two groups (*P* > 0.05), the fixation segments in CPS group (3.5 ± 1.1) were less than that in LMS group (4.2 ± 0.7) (*P* = 0.037). The accuracy of CPS insertion was evaluated based on postoperative CT. Of all the 118 CPSs, 83 (70.3%) were defined as Grade 0; 27 (22.9%) as Grade 1; eight (6.8%) as Grade 2; and none as Grade 3. CPS malposition rate in this study was 6.8%. In this study, there was no direct intraoperative or postoperative complication caused by CPS or LMS insertion. All the operations were successfully completed in two groups. One of the patients in LMS group presented cerebrospinal fluid leak caused by bone fragment broken of the dural sac, which led to delayed incision healing. CPS group and LMS group both had two patients who suffered pulmonary infection after surgery. A total of 78.6% of the patients showed evidence of neurologic recovery. Satisfactory reduction was achieved in all cases and maintained throughout the follow‐up duration.

**Conclusion:**

In the treatment of lower cervical spine fracture‐dislocation, cervical pedicle screw insertion with O‐arm navigation is a safe and effective method for posterior fixation.

## Graphical abstract

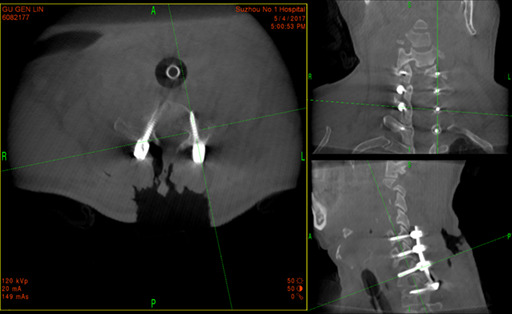

## Introduction

Nowadays, the fracture‐dislocation of lower cervical spine, a severe traumatic lesion, is becoming more and more frequent with the development of social economy^1^. The prognosis of this injury is poor, which often leads to severe cervical instability and spinal cord injury with high fatality and disability rate^2^, ^3^. According to previous research, spinal cord injury was observed in 37%–100% of cervical facet dislocation cases^4^. It is of great importance to reconstruct the cervical stabilization, restore the cervical vertebrae alignment, enhance bony fusion, and decompress the spinal canal completely^3^. However, the treatment of fracture‐dislocation of cervical spine remains controversial now. Several studies regarding classifications and treatments have been carried out, but there is no universally accepted and perfect internal fixation system for the treatment of cervical fracture and dislocation^5^.

As compared to other traditional fixation techniques, like lateral mass screw, cervical pedicle screw (CPS) provides greater stability and distinct clinical advantage, making it the best biomechanical system for posterior segmental fixation^6^, ^7^, ^8^, ^9^. However, the use of CPS has not been well‐adopted because of significant concerns for neurovascular injury associated with CPS malposition^10^, ^11^, ^12^. Nakashima *et al*. reported that five patients suffered neurovascular injury complications due to cervical pedicle screw misplacement, including nerve root injury by pedicle screw in three patients and vertebral artery injury in two patients^13^. Hojo *et al*. showed the total perforation of cervical pedicle screws inserted by freehand technique was 14.8% (158/1065), two patients suffered vertebral artery injury, and six patients had root injuries on account of the CPS malposition in this study^14^. Thus, the accurate application of CPS is very challenging for spine surgeons. How to improve the accuracy of cervical pedicle screw has attracted wide attention.

Various methods have been applied to assist cervical pedicle screw placement for improving accuracy, including image‐assisted navigation system and individualized 3D printing navigation template^15^. According to the reports, the original navigation system did improve the accuracy of the cervical pedicle screw insertion, but it did not satisfy our hunger for CPS placement accuracy. Primary image‐assisted navigation techniques mainly depend on preoperative computed tomography (CT) scans. This technique improves CPS insertion accuracy, but navigation error tends to occur due to the registration error. This defect contributed to the development of the intraoperative navigation with automatic registration^15^, ^16^, ^17^, ^18^. Hence, development of an intraoperative navigation system is urgent.

The state‐of‐art image‐assisted navigation system is an intraoperative full‐rotation, multi‐dimensional image system (O‐arm) which allows immediate real‐time image guidance. The O‐arm‐based navigation system has been reported to have high accuracy and safety in thoracic‐lumbar spine surgery^19^. However, few studies have reported on the outcome of cervical pedicle screw fixation with O‐arm navigation in the lower cervical spine surgery^20^, ^21^. Compared to conventional lateral mass screw, the advantage of CPS in treating lower cervical fracture‐dislocation remains unclear. Therefore, the purpose of our study has been divided into the following three aspects: (i) investigating the safety and efficiency of CPS insertion with O‐arm navigation in the treatment of lower cervical fracture‐dislocation; (ii) evaluating the advantages and disadvantages of CPS in comparison with lateral mass screw insertion in treating lower cervical fracture‐dislocation; and (iii) detecting the accuracy of CPS placement with O‐arm navigation in cervical spine.

## Materials and Methods

### The Inclusion and Exclusion Criteria

The inclusion criteria were as follows: (i) patients had single segment cervical spine fracture‐dislocation and were followed up for more than 6 months; (ii) patients who underwent CPS fixation with O‐arm navigation or lateral mass screw fixation only through posterior approach; (iii) Subaxial Cervical Spine Injury Classification (SLIC) scale, American Spinal Injury Association (ASIA) score, Japanese Orthopaedic Association (JOA) score, and complications were used for evaluation of the severity; and (iv) all the patients' outcomes were documented.

The exclusion criteria were as follows: (i) patients had obvious cervical disc fragments in the canal compressed spinal cord; (ii) patients had other severe disease that may affect clinical outcome such as severe cerebral trauma; (iii) patients had multilevel cervical spine fracture‐dislocation; and (iv) there were other systemic diseases that affected the number of fixation segments.

### Grouping

**Fig. 1:**
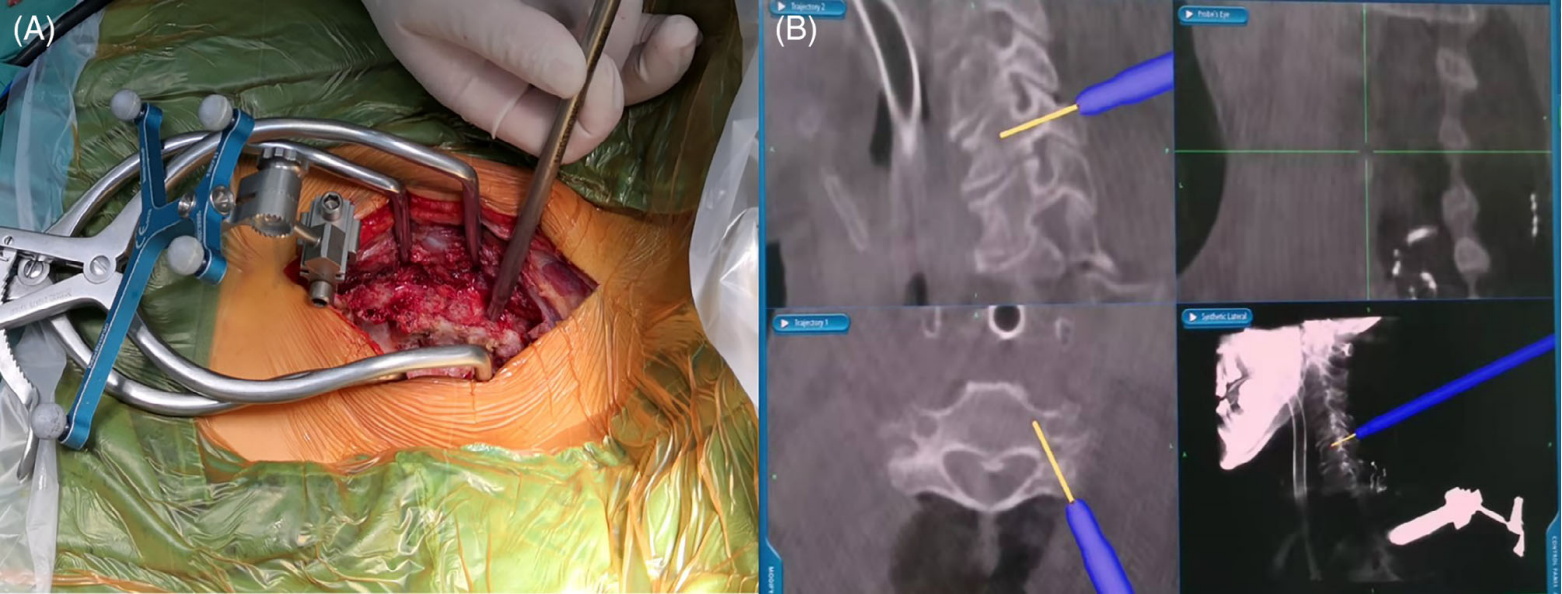
Cervical pedicle screw (CPS) placement with O‐arm navigation during operation. (A) The navigation reference frame was attached to the spinous process. (B) Intraoperative 3D image when CPS was inserted with O‐arm navigation.

Between August 2015 and August 2019, 42 consecutive lower cervical fracture‐dislocation patients (37 males and five females) were included in this study. These patients were divided into two groups, CPS group (including 22 patients who were treated by CPS fixation with O‐arm/stealth navigation system) (Fig. 1) and LMS (lateral mass) group (including 20 patients who were treated by conventional lateral mass screw fixation).

CPS group: the patients' average age at surgery was 52.2 years (range, 29–78 years), follow‐up ranged from 6 to 44 months. Injury mechanism: nine patients of lower cervical spine fracture‐dislocation suffered from traffic accidents, eight cases by falling from high places, two cases by heavy object crashes, three cases by other reasons. Injury location: one patient had C_3−4_ fracture‐dislocation, three patients located at C_4−5_, three cases located at C_5−6_, eight patients located at C_6−7_, and seven patients located at C_7_‐T_1_.

LMS group: the average age of patients at surgery was 49.5 years (range, 22–66 years), follow‐up ranged from 6 to 40 months. Injury mechanism: five patients of lower cervical spine fracture‐dislocation suffered from traffic accidents, nine cases by falling from high places (FHP), three cases by heavy object crashes (HOC), three cases by other reasons. Injury location: two patients have C_3−4_ fracture‐dislocation, four patients located at C_4−5_, five cases located at C_5−6_, six patients located at C_6−7,_ and three patients located at C_7_‐T_1_.

All the patients presented various degrees of spinal cord injury and their surgeries were performed by one single group surgeon. This study has been approved by the ethics committee of the First Affiliated Hospital of Soochow.

### Surgical Procedure

CPS group: After intubation under general anesthesia, the patient was placed prone on the carbon‐fiber table and cervical spine was maintained in neutral position by continuous skull traction. A standard posterior midline incision was made, the paraspinous muscles were dissected, the lateral mass and its lateral margin were exposed sufficiently. The navigation reference frame was attached to the spinous process of the vertebrae one level caudal to the most distal pedicle screw or cephalad to the most proximal pedicle screw to be placed by navigation, and 3D image data was obtained by O‐arm. After the navigation was ready, an entry point was determined. The trajectory of each screw was visualized on the Stealth Station's axial and sagittal images. After pedicle screws of appropriate diameters were inserted, intraoperative O‐arm scan was done again to confirm the accuracy of CPS, then completed reduction. Suitable rods were selected, placed, and caps were screwed tightly after reduction. At last, O‐arm scan was done again to affirm the reduction. Laminectomy or laminoplasty was performed if necessary.

LMS group: After general anesthesia, the patient was placed in a prone position under axial traction. A standard posterior middle incision was made. After exposure, a distraction force was applied between the spinous processes. For those cases that reduction could not be achieved because of locked facets, the tip of the superior articular process of the distal segment would be resected to release the facets. Lateral mass screws were inserted by Magerl technology. After the application of suitable rods, compression would be done according to spinal cord injury.

### Postoperative Management

After the surgery, we removed the skull traction and used the cervical collar instead. For the patients with incomplete spinal cord injury, sitting and walking was allowed on the first day after operation.

### Outcome Measures

#### Japanese Orthopaedic Association (JOA) Score System

The JOA score was used to assess neurological function before and after surgery, including upper limb movement function (4 points), lower limb motor function (4 points), sensory (6 points) and bladder function (3 points). The total score was 17 points, which was considered normal.

#### American Spinal Injury Association (ASIA) Grade

ASIA grade was applied to evaluate the spinal cord injury before and after surgery. ASIA grade was divided into grade A–E. A: complete neurological injury with no motor and sensory function. B: complete sensory loss with preserved sensation. C: preserved no functional motor. D: preserved functional motor. E: normal muscle strength and sensation.

#### Subaxial Cervical Spine Injury Classification

SLIC scale system was used to evaluate and describe any given injury to the subaxial cervical spine and guide treatment. The score was according to injury morphology, discoligamentous complex and neurologic status. If total score >4, surgery is recommended, if total score <4, conservative treatment is most likely indicated.

#### Cervical Pedicle Screw (CPS) Placement Accuracy

The accuracy of CPS position was evaluated by reviewing postoperative CT scans. The classification is as follows: grade 0: if no cortical breach; grade 1: if a cortical perforation is present and the screw protrudes up to 2 mm; grade 2: if the cortical perforation is >2 mm; and grade 3: screws placed outside of the pedicle, CPS malposition was defined as any screw placement rated grade 2 or above^22^, ^23^.

The fusion rate and complications were also recorded for the two groups.

### Statistical Analysis

SPSS 19.0 statistical analysis software (IBM, New York, NY) was used for all the statistical analyses. The measurement data (age, SLIC score, Number of fixation segments, intraoperative blood loss, operation time, follow‐up time, ASIA score, JOA score) were expressed as mean ± SD (x ± s). Two independent sample *t* tests were utilized for measurement data comparison. The Mann–Whitney U test and chi square test were used to compare categorical variables (gender, injury mechanism, injury location). *P* < 0.05 indicated a statistically significant difference.

## Results

### General Results

Between the CPS group and LMS group, there was no statistically significant difference in gender, age, injury mechanism, injury location and follow‐up time (Table 1, *P* > 0.05). The mean operation time in LMS group (204 ± 89 min) is shorter than that in CPS group (241 ± 85 min) and the mean intraoperative blood loss in LMS group (311 ± 127 ml) is greater than that in CPS group (327 ± 120 ml), but these two differences were not statistically significant (Table 1, *P* > 0.05). However, the number of fixation segments in LMS group (4.2 ± 0.7) is significantly more than that in CPS group (3.5 ± 1.1) (Table 1, *P* = 0.037).

**TABLE 1:** Clinical parameters comparison in LMS and CPS group

Clinical parameter	LMS group	CPS group	*t or X* ^*2*^ *value*	*P* Value
Age	49.5 ± 14.2	52.2 ± 14.1	−0.636	0.528
Gender			0.579	0.566
Male	17	20		
Female	3	2		
Injury mechanism			1.309	0.727
Traffic accident	5	9		
FHP	9	8		
HOR	3	2		
Other reasons	3	3		
Injury location			3.613	0.461
C_3,4_	2	1		
C_4,5_	4	3		
C_5,6_	5	3		
C_6,7_	6	8		
C_7_,T_1_	3	7		
Preoperative SLIC score	7.5 ± 0.9	7.3 ± 1.2	0.539	0.593
Number of fixation segments	4.2 ± 0.7	3.5 ± 1.1	2157	0.037
Intraoperative blood loss (ml)	311 ± 127	327 ± 120	−0.44	0.662
Operation time (min)	204 ± 89	241 ± 85	−1.356	0.183
Follow‐up time (m)	16 ± 12	16 ± 11	−0.048	0.962
ASIA score				
Preoperative	1.3 ± 1.1	1.3 ± 1.2	0.076	0.940
Final follow‐up	2.1 ± 1.4	2.1 ± 1.5	−0.082	0.935
Improvement	0.8 ± 0.5	0.9 ± 0.5	−0.416	0.679
JOA score				
Preoperative	5.3 ± 4.5	5.1 ± 4.8	0.079	0.938
Final follow‐up	8.2 ± 5.5	8.3 ± 6.1	−0.066	0.948
Improvement	3.0 ± 1.8	3.2 ± 2.4	−0.355	0.724

*Note*: Data were expressed as mean ± SD (x ± s). *P* < 0.05 indicated a statistically significant difference.

Abbreviations: ASIA, American Spinal Injury Association; CPS, cervical pedicle screw; FHP, falling from high places; HOC, heavy object crashes; JOA, Japanese Orthopaedic Association; LMS, lateral mass screw; SLIC, Subaxial Cervical Spine Injury Classification.

### Functional Outcomes

Between the CPS group and LMS group, there was no statistically significant difference in preoperative and final follow‐up JOA score (Table 1, *P* > 0.05). Moreover, JOA score improved from 5.3 to 8.2 and 5.1 to 8.3 in LMS group and CPS group, respectively, but no statistical difference was found between the two groups (Table 1, *P* > 0.05).

The ASIA grade (A–E) was converted to a numerical quantization score (0–4). Between the CPS group and LMS group, there was no statistically significant difference in preoperative ASIA grade and SLIC score (Table 1, *P* > 0.05). At the last follow‐up, ASIA score improved from 1.30 to 2.10 and 1.27 to 2.14 in LMS and CPS group, respectively, which was also not statistically different between the two groups (Table 1, *P* > 0.05).

### Accuracy of Cervical Pedicle Screw (CPS) Placement

One hundred and ten CPSs were inserted into the cervical vertebrate pedicles. The number of screws inserted at each level was as follows: C_3,_ four; C_4_, 16; C_5_, 26; C_6_, 36; and C_7_, 36. Evaluations of the position of the inserted CPSs on postoperative CT scans are featured in Table 2. Of the 118 CPSs, 83 (70.3%) were defined as Grade 0; 27 (22.9%) as Grade 1; eight (6.8%) as Grade 2; and none as Grade 3. CPS malposition rate in this study was 6.8% (Table 2).

**TABLE 2:** Accuracy of CPS placement in cervical spine

Spine	No. of CPSs	Grade 0	Grade 1	Grade 2	Grade 3	Grade 0–1(%)
C_3_	4	2	2	0	0	100
C_4_	16	6	7	3	0	81.3
C_5_	26	18	5	3	0	88.5
C_6_	36	29	5	2	0	94.4
C_7_	36	28	8	0	0	100
Total	118	83	27	8	0	93.2

Abbreviation: CPS, cervical pedicle screw.

### Complications

In this study, there was no direct intraoperative or postoperative complication, including injury to the vertebral artery, spinal cord, or nerve root, caused by CPS or LMS insertion. All the operations were successfully completed in two groups. One of the patients in LMS group presented cerebrospinal fluid leak caused by bone fragment broken of the dural sac, which led to delayed incision primary healing. CPS group and LMS group both had two patients who suffered pulmonary infection after surgery. A total of 78.6% of the patients showed evidence of neurologic recovery (Table 3). All the patients had achieved solid bony fusion at the last follow‐up.

**TABLE 3:** Neurological evaluation with the ASIA Impairment Scale in two groups

Preoperative (LMS group/CPS group)	Final follow‐up (LMS group/CPS group)				
	A	B	C	D	E
A	4/4	1/3			
B			8/7	1/1	
C			1/0	0/1	
D					5/6

Abbreviations: ASIA, American Spinal Injury Association; CPS, cervical pedicle screw; LMS, lateral mass screw.

### Illustrative Case in Cervical Pedicle Screw (CPS) Group

**Fig. 2:**
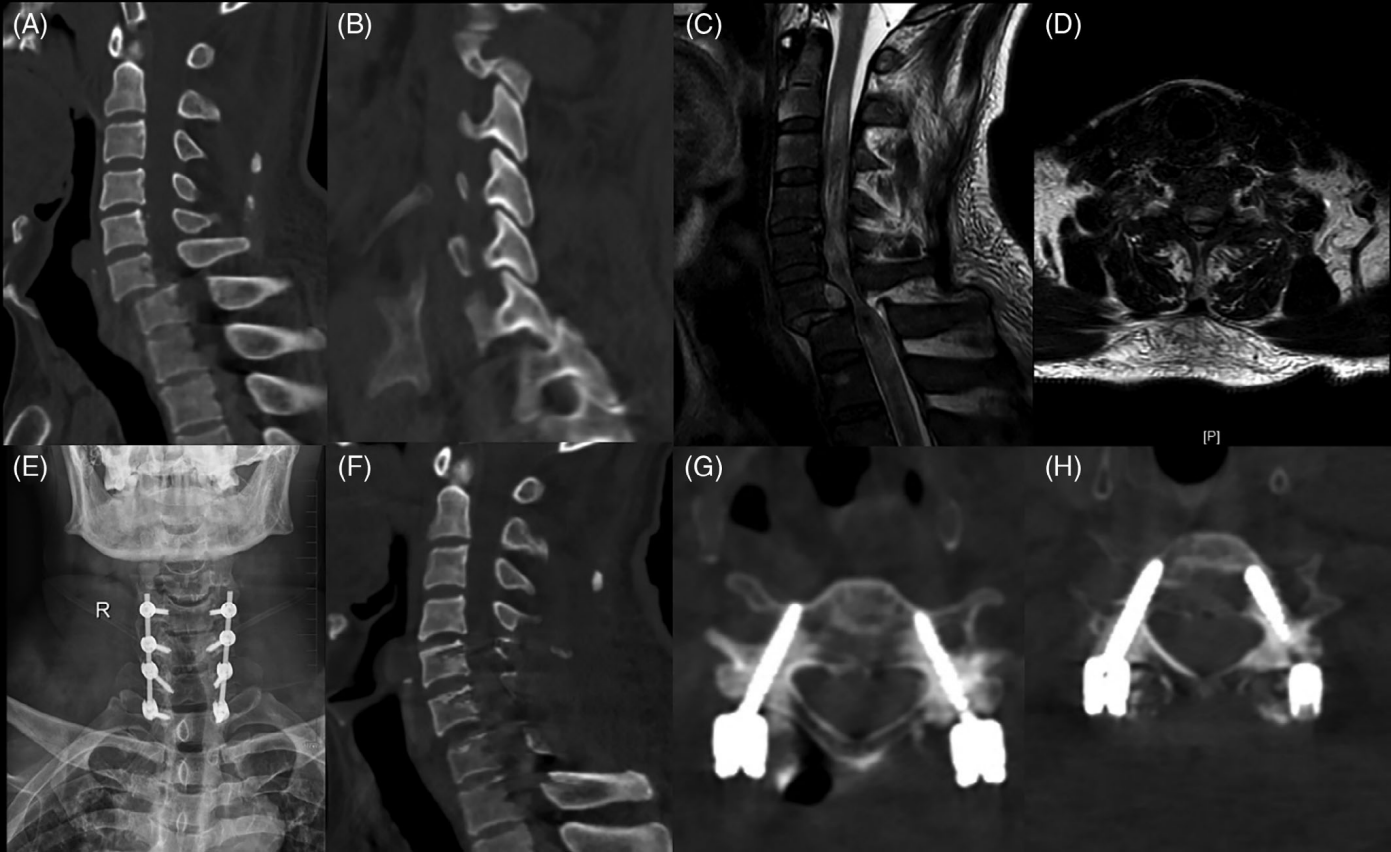
A 55‐year‐old male patient with C_6,7_ fracture‐dislocation undergoing cervical pedicle screw (CPS) fixation treatment with O‐arm navigation. (A‐D) Preoperative CT and MRI showed C_6,7_ fracture‐dislocation with bilateral locked facets and the spinal cord was obviously compressed. (E–H) Postoperative X‐ray and CT showed complete reduction and decompression for the cervical spine and the CPSs were all placed accurately in cervical pedicles.

**Fig. 3:**
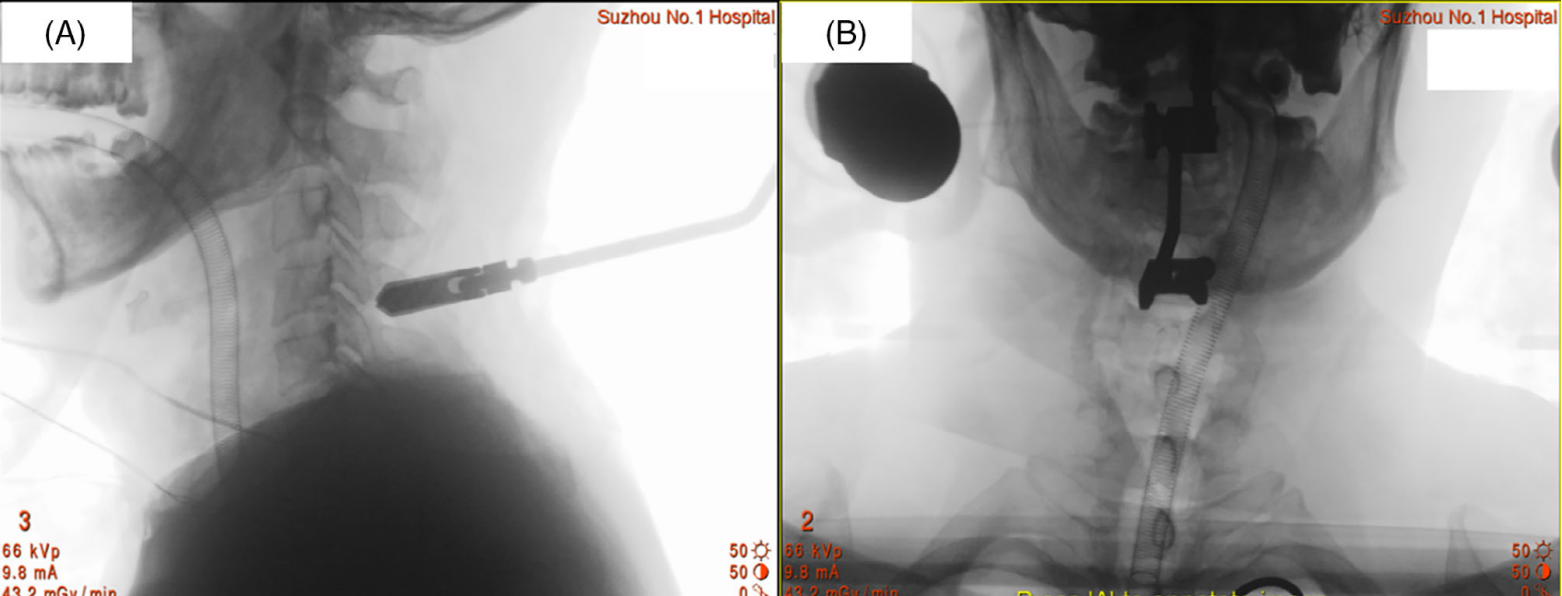
Intraoperative image showed navigation reference frame was attached to the spinous process of C_4_ vertebrate. (A) Intraoperative lateral film of cervical spine demonstrated that navigation reference frame was attached to spinous process of C_4_ vertebrate. (B) Intraoperative anteroposterior film of cervical spine indicated location of navigation reference frame in cervical spine.

**Fig. 4:**
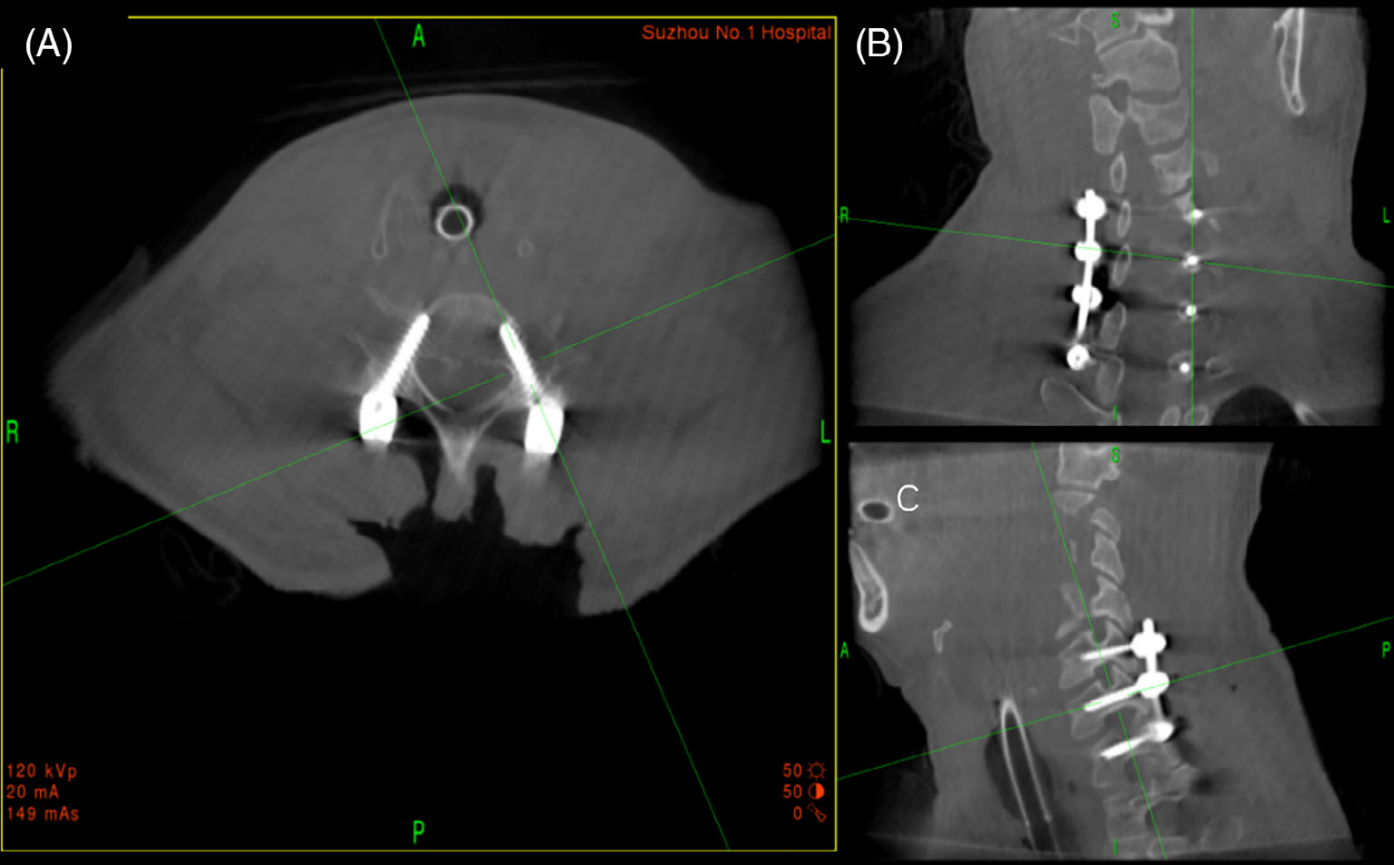
Intraoperative 3D image data obtained by O‐arm showing cervical pedicle screw (CPS)s were accurately inserted in the cervical pedicle. (A) Intraoperative cross‐sectional CT of cervical spine after CPS insertion. (B) Intraoperative cervical spine coronal CT after CPS insertion. (C) Intraoperative cervical spine sagittal CT after CPS placement.

A 55‐year‐old male patient suffered falling accident, sustained a C_6−7_ bilateral facet joint dislocation. Preoperative neurological status was ASIA‐A. Radiographic results before operation showed cervical spine fracture‐dislocation at C_6−7_. Posterior fixation, decompression, and fusion of C_6−7_ was conducted by using pedicle screw system with O‐arm navigation. Neurological improvement was not observed after surgery, although postoperative CT showed full decompression in spinal canal (Figs. 2, 3, 4).

## Discussion

### Advantage of Cervical Pedicle Screw (CPS) Insertion through Posterior Approach in Treatment of Cervical Spine Fracture‐Dislocation

Fracture‐dislocation of lower cervical spine is one of the most devastating cervical injuries with complete anterior and posterior column instability. A number of methods have been described for treatment of this injury. Nevertheless, the approach for the disorder is still a controversial topic^3^, ^24^, ^25^. Compared to anterior approach, posterior approach is easier in reduction for the cervical spine fracture‐dislocation with locked facets^26^. Furthermore, posterior approach has more advantage in controlling the risk as surgical site infection in the patient undergoing endotracheal. The most common posterior instrument‐lateral mass screw was once thought to be relatively reliable. However, it is only involved with posterior column fixation, and could not be used in the presence of osteoporosis or lateral mass fracture^27^. As reported in previous research, CPSs are stronger in a three‐column injury model and can offer the best pull‐out resistance of all available posterior fixation techniques^9^, ^10^, ^11^, ^12^, ^13^, ^14^, ^15^, ^16^, ^17^, ^18^, ^19^, ^20^, ^21^, ^22^, ^23^, ^24^, ^25^, ^26^, ^27^, ^28^. Besides, CPS can obviate the requirement for anterior–posterior procedures in cases with high risk of pseudarthrosis or construct failure^29^. As we know, an ideal internal fixation system should provide adequate stability and enhance bone fusion because of the extreme instability associated with fractures and dislocations. These advantages make CPS extremely useful for the reduction of spine fracture‐dislocation. They may play an important role in facilitating earlier mobilization and rehabilitation.

### Safety and Accuracy of Cervical Pedicle Screw (CPS) Insertion with O‐Arm Navigation

Pedicle screws have not been widely used in cervical spine surgery despite of those benefits. Spine surgeons showed significant concerns for the serious neurovascular complications related to the pedicle screw misplacement, such as injury to the vertebral artery, spinal cord, or nerve roots^30^. Abumi *et al*. reported three neurovascular complications directly attributed to screw insertion by free‐hand technique with fluoroscopy^31^, while Neo *et al*. showed that the percentage of CPS malposition was up to 29.1%^32^. Uehara *et al*. reported that rate of CPS perforations was up to 20% (116/579)^33^, even if preoperative 3D CT‐based navigation system was used. In contrast, the latest image‐assisted navigation (O‐arm/stealth navigation system) has been developed to improve the accuracy of CPS insertion. Ishikawa *et al*. showed that 97% of 108 CPSs was accurately inserted with O‐arm navigation^16^. Consistently, our study demonstrated that the misplacement rate of CPS by using intraoperative O‐arm navigation system is just 6.8% (8/118) and all the CPSs are clinically safe without neurovascular injury. CPS systems can provide adequate three‐dimensional stability, and now can be safely placed with the assistance of O‐arm navigation.

### Efficacy of Cervical Pedicle Screw (CPS) and LMS Fixation in the Treatment of Lower Cervical Spine Fracture‐Dislocation

In this study, we performed the CPS fixation with CT‐guided navigation system in the treatment of lower cervical spine fracture‐dislocation. Although operation time and intraoperative blood loss in traditional LMS group is less than that in CPS group, the difference is not statistically significant. However, the number of fixation segments in CPS is significantly less than that in LMS group (*P* < 0.05), which may partly reflect less surgical trauma in CPS group. Moreover, our study showed satisfactory reduction in all cases that was maintained throughout the follow‐up duration. According to the JOA score and ASIA impairment scale, most of the patients from the two groups presented different degrees of progress in spinal cord function over time, there is no significant difference in JOA or ASIA sore improvement between the two groups. There was no direct intraoperative or postoperative complication, such as injury to the vertebral artery, spinal cord, or nerve root, caused by CPS or LMS insertion. In short, CPS system may present a little less surgical trauma than LMS system, both CPS and LMS are effective in the treatment of cervical spine fracture‐dislocation.

### How to Insert Cervical Pedicle Screw (CPS) Accurately with O‐Arm Navigation

O‐arm navigation can obviously improve the accuracy of pedicle screw insertion, but cervical spine navigation surgery is different from thoracic and lumbar navigation surgery. Fracture‐dislocation, together with inherent high mobility, makes cervical spine extremely unstable. Based on our experience, when navigation reference frame was attached to the spinous process of the vertebrate, the surgeons should be aware of the movement of the reference frame away from surgical region, which can lead to the inaccurate navigation. To avoid this problem, the cervical spine should be maintained in neutral position with continuous skull traction all the way during the operation. As respiration causing movement during image acquisition can also lead to significant inaccuracy, the anesthesiologist needs to hold the patient's respiration during image acquisition, and the surgeon should reconfirm the navigation accuracy prior to CPS insertion. CPSs should be planted from far to near as to the navigation reference frame. Drilling the hole by electric drill instead of freehand may decrease the deformation of cervical spine, so as to improve the navigation accuracy.

### Limitations

The drawback of the current study is the limited number of cases enrolled, as the low incidence of lower cervical spine fracture‐dislocation. More patients will be covered in future study. Meanwhile, the amount of radiation to the patient exposed to radiation cannot be exactly quantified. Further investigation should ideally measure the radiation dose exposed to patients. Furthermore, a comparative study will be carried out in the future to confirm the validity of O‐arm's benefits over other imaging systems.

### Conclusion

Although O‐arm navigation has its own limitations, such as steep learning curve, it can allow for immediate evaluation of screw position and significantly improve the accuracy and safety of CPS insertion. Compared to LMS, application of CPS may show less surgical trauma in treating cervical fracture‐dislocation. Placement of CPS using O‐arm navigation is a very safe and effective method for posterior stabilization of lower cervical spine fracture‐dislocation.
